# Supplementary material for: Intravenous gene therapy improves lifespan and clinical outcomes in feline Sandhoff Disease
Source: bioRxiv. 2024 Nov 18:2024.11.15.623838. Preprint. [Version 1] doi: 10.1101/2024.11.15.623838 (PMC11601349; doi:10.1101/2024.11.15.623838)
Supplement: Supplement 1 — Fig S1. Spinal cord compression in AAV-treated cat Fig S2. Ultrasound elastography Fig S3. Specific activity of Total Hex, Bgal, and Mann lysosomal enzymes in the CNS Fig S4. Total sialic acid storage products in AAV-treated cats Fig S5. Histopathological storage lesions in neuronal cell bodies and hepatocytes are partially corrected by AAV treatment Fig S6. AAV vector biodistribution Fig S7. Serum antibody titers in AAV-treated cats [file NIHPP2024.11.15.623838v1-supplement-1.pdf]

bioRxiv preprint doi: https://doi.org/10.1101/2024.11.15.623838; this version posted November 18, 2024. The copyright holder for this preprint (which was not certified by peer review) is the author/funder, who has granted bioRxiv a license to display the preprint in perpetuity. It is made available under aCC-BY 4.0 International license.

Supplementary Materials and Methods

Immunohistochemistry detailed protocol

- General protocol:
    1. Block: IP FLX Peroxidase, 5 minutes
    2. Block: IP FLX Background Punisher, 5 minutes
    3. Primary antibody: Diluted in Da Vinci Green Diluent
    4. Secondary antibody: MACH 2 Rabbit HRP-Polymer, 30 minutes
    5. Chromogen: Betazoid DAB, 5 minutes
    6. Counter-stain: Tacha's Auto Hematoxylin, 3 minutes
- Buffer wash throughout: TBS Auto Wash Buffer
- GFAP primary antibody: 1:200 dilution, 20 minutes
- Iba-1 primary antibody: 1:750 dilution, 30 minutes
- Olig2 primary antibody: Abcam, 1:200 dilution, 30 minutes

Slides dried overnight at room temperature and were coverslipped the following day.

QuPath workflow

The first step in the analysis of each image was to manually input the magnification as 20x and pixel length as 0.2 x 0.2 µm, since that information was not imported automatically with the metadata attached to each slide. Second, a stain vector was applied, if needed, to optimize the software's differentiation between hematoxylin and DAB or LFB (stain vectors provided below). Third, regions of interest (parietal cortex white matter/gray matter, thalamus, cerebellar cortex white matter/gray matter, DCN, brainstem) were hand-drawn using the brush and wand tools. For LFB-stained slides, determination of white/gray matter boundaries during this step was performed while viewing the hematoxylin-only "channel" to avoid bias introduced by LFB staining. Fourth, the algorithm was applied and the resulting data manually recorded in a Microsoft Excel file.

QuPath algorithms

- Olig2
    - Stain vectors:
        - Hematoxylin: 0.739 0.538 0.405
        - DAB: 0.452 0.589 0.67
        - Background: 240 240 240
    - Cell detection:
        - Detection image: Optical density sum
        - Requested pixel size: 0.2 µm

bioRxiv preprint doi: https://doi.org/10.1101/2024.11.15.623838; this version posted November 18, 2024. The copyright holder for this preprint (which was not certified by peer review) is the author/funder, who has granted bioRxiv a license to display the preprint in perpetuity. It is made available under aCC-BY 4.0 International license.

        - Background radius: 10 µm
        - Median filter radius: 0 µm
        - Sigma: 1.0 µm
        - Minimum area: 6 µm^2
        - Maximum area: 400 µm^2
        - Threshold: 0.25
        - Maximum background intensity: 2.0
        - Split by shape: true
        - Exclude DAB: false
        - Cell expansion parameters: 0 µm
        - Include cell nucleus: true
        - Smooth boundaries: true
        - Make measurements: true
    - Object classification
        - Channel filter: DAB
        - Measurement: DAB OD Mean
        - Threshold: 0.08
        - Above: Positive
        - Below: Negative
- NeuN
    - Stain vector (faint hematoxylin)
        - Hematoxylin: 0.50236 0.60145 0.6212
        - DAB: 0.31918 0.51223 0.79734
        - Background: 223 223 220
    - Stain vector (strong hematoxylin)
        - Hematoxylin: 0.607 0.628 0.488
        - DAB: 0.401 0.573 0.715
        - Background: 221 223 221
    - Cell detection (faint hematoxylin)
        - Detection image: Optical density sum
        - Requested pixel size: 0.2 µm
        - Background radius: 18 µm

bioRxiv preprint doi: https://doi.org/10.1101/2024.11.15.623838; this version posted November 18, 2024. The copyright holder for this preprint (which was not certified by peer review) is the author/funder, who has granted bioRxiv a license to display the preprint in perpetuity. It is made available under aCC-BY 4.0 International license.

    - Median filter radius: 0 µm
    - Sigma: 1.5 µm
    - Minimum area: 6 μm^2
    - Maximum area: 600 μm^2
    - Threshold: 0.3
    - Maximum background intensity: 2.0
    - Split by shape: false
    - Exclude DAB: false
    - Cell expansion parameters: 3 µm
    - Include cell nucleus: true
    - Smooth boundaries: true
    - Make measurements: true
  - Cell detection (strong hematoxylin)
    - Same as faint hematoxylin except threshold = 0.4
  - Object classification (faint hematoxylin) #1
    - Object filter: Detections (all)
    - Channel filter: Hematoxylin
    - Measurement: Nucleus Hematoxylin OD Mean
    - Above: Negative
    - Below: Positive
  - Object classification (faint hematoxylin) #2
    - Object filter: Detections (all)
    - Channel filter: DAB
    - Measurement: Nucleus DAB OD Max
    - Above: Positive
    - Below: Negative
  - Object classification (strong hematoxylin)
    - Object filter: Detections (all)
    - Channel filter: DAB
    - Measurement: Nucleus DAB OD Mean
    - Threshold: 0.15
    - Above: Positive
    - Below: Negative

bioRxiv preprint doi: https://doi.org/10.1101/2024.11.15.623838; this version posted November 18, 2024. The copyright holder for this preprint (which was not certified by peer review) is the author/funder, who has granted bioRxiv a license to display the preprint in perpetuity. It is made available under aCC-BY 4.0 International license.

    - Stain vector (for strong-hematoxylin slides, cerebellar cortex only)
        - Hematoxylin: 0.72 0.641 0.265
        - DAB: 0.435 0.568 0.699
        - Background: 217 220 220
- Calbindin
    - Stain vector: Default
    - Thresholder: same as NeuN thresholder, except Threshold = 0.30
- GFAP
    - Stain vector: Default
    - Thresholder (for strong-hematoxylin slides)
        - Resolution: Very high
        - Channel: DAB
        - Prefilter: Gaussian
        - Smoothing sigma: 0
        - Threshold: 0.20
        - Above threshold: Positive
        - Below threshold: Negative
        - Region: Any annotations
- Iba-1
    - Stain vector: Default
    - Thresholder: same as GFAP thresholder, except Threshold = 0.15
- LFB
    - Stain vector
        - Hematoxylin: 0.644 0.722 0.251
        - LFB: 0.777 0.516 0.359
        - Background: 238 238 236
    - Thresholder:
        - Channel: LFB
        - Above threshold: Positive
        - Below threshold: Negative

bioRxiv preprint doi: https://doi.org/10.1101/2024.11.15.623838; this version posted November 18, 2024. The copyright holder for this preprint (which was not certified by peer review) is the author/funder, who has granted bioRxiv a license to display the preprint in perpetuity. It is made available under aCC-BY 4.0 International license.

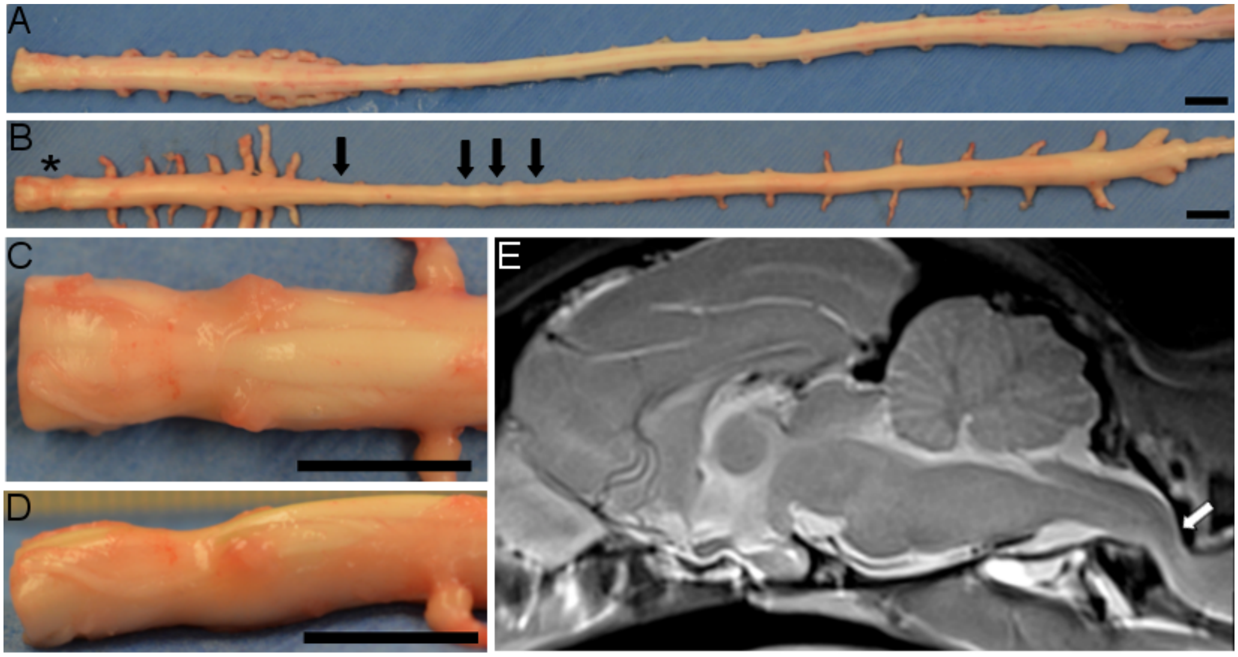

Figure S1. Spinal cord compression in AAV-treated cat. (A) Spinal cord of normal cat at necropsy. (B) Spinal cord of SD cat #52 of the high-dose cohort at necropsy (9.6 months old) with multiple compression lesions. Black arrows indicate sites of compression at T3 and T7-T9. * indicates most severe site of compression at C1, which is also depicted in (C-E). (C) Magnified view of dorsal surface of C1 compression lesion. (D) Magnified view of left lateral surface of C1 compression lesion. (E) Sagittal view of T2-weighted MRI on midline. White arrow indicates where the thin bright line of the CSF-filled central canal is interrupted at C1 due to spinal cord compression. Scale bar: 1cm.

bioRxiv preprint doi: https://doi.org/10.1101/2024.11.15.623838; this version posted November 18, 2024. The copyright holder for this preprint (which was not certified by peer review) is the author/funder, who has granted bioRxiv a license to display the preprint in perpetuity. It is made available under aCC-BY 4.0 International license.

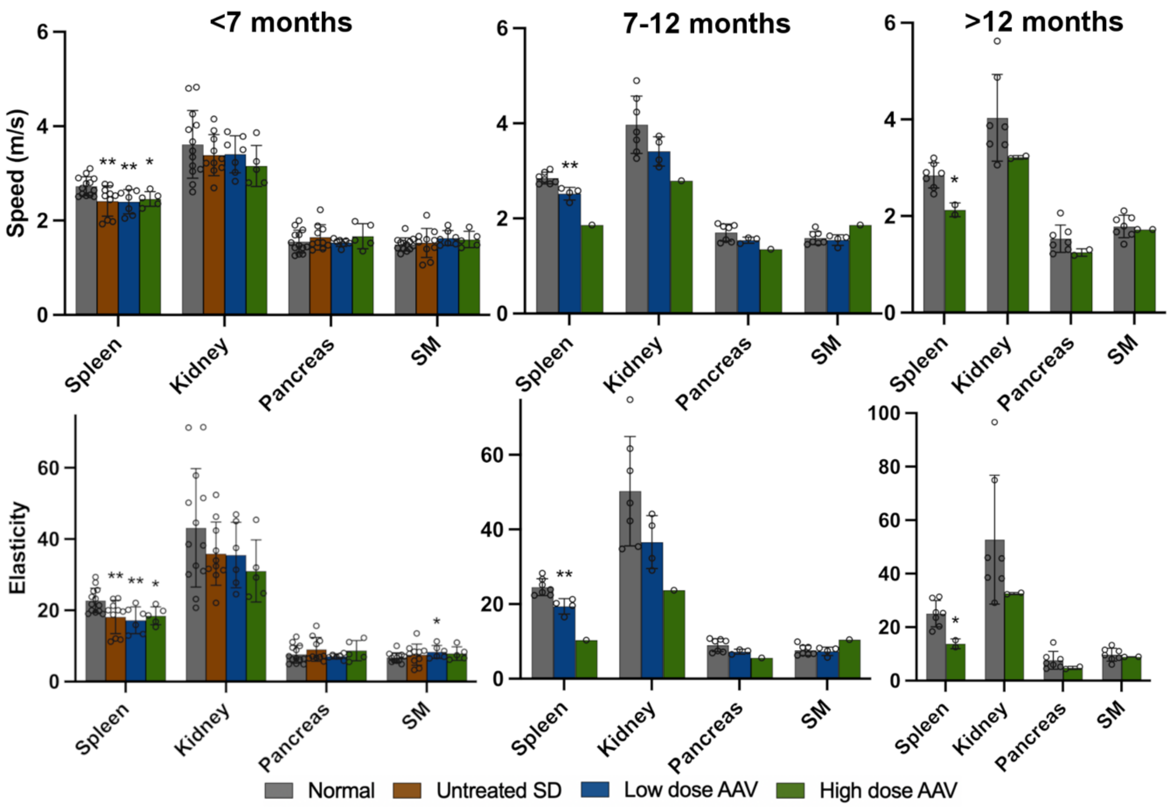

Figure S2. Ultrasound elastography. Shear wave ultrasound elastography results in the spleen, kidney, pancreas and skeletal muscle (SM). *p<0.05, **0.01>p>0.001 vs. age-matched normal cats.

bioRxiv preprint doi: https://doi.org/10.1101/2024.11.15.623838; this version posted November 18, 2024. The copyright holder for this preprint (which was not certified by peer review) is the author/funder, who has granted bioRxiv a license to display the preprint in perpetuity. It is made available under aCC-BY 4.0 International license.

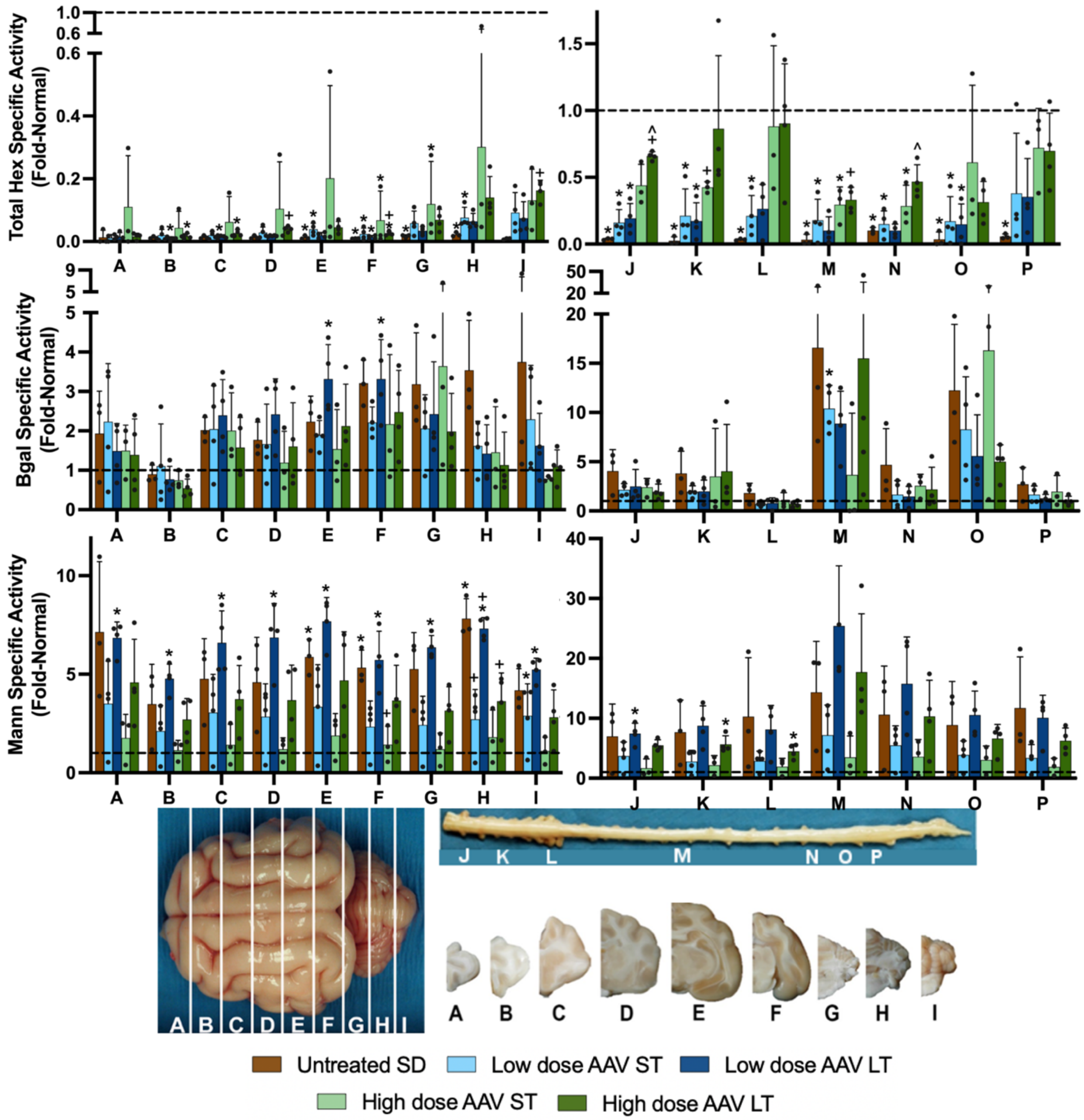

Fig S3. Specific activity of Total Hex, Bgal, and Mann lysosomal enzymes in the CNS
Specific activity of combined Hex isozymes (Total Hex) in the brain and spinal cord increases with AAV treatment in a dose-dependent fashion. Specific activity of other lysosomal hydrolases ß-galactosidase (Bgal) and α-mannosidase (Mann) increase to above-normal levels in SD cats in a compensatory attempt to reduce the abnormally high amount of storage material. Reduction of Bgal and Mann levels in AAV-treated cats corresponds with increases in HexT activity. * indicates $p<0.05$ compared to age-matched normal cats, + indicates $p<0.05$ compared to untreated SD cats, and ^ indicates $p<0.05$ compared to low dose at same age.

bioRxiv preprint doi: https://doi.org/10.1101/2024.11.15.623838; this version posted November 18, 2024. The copyright holder for this preprint (which was not certified by peer review) is the author/funder, who has granted bioRxiv a license to display the preprint in perpetuity. It is made available under aCC-BY 4.0 International license.

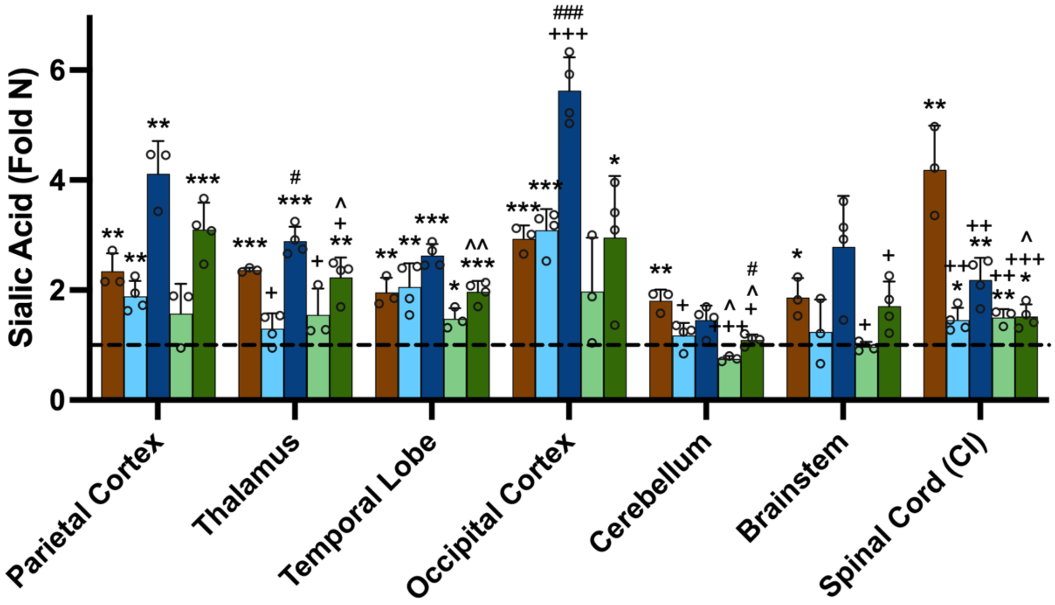

Fig S4. Total sialic acid storage products in AAV-treated cats. Total sialic acid (SA) is significantly increased above normal in 5/7 CNS regions examined. CI: cervical intumescence. *p<0.05, **0.01>p>0.001, ***0.001<p<0.0001 vs. age-matched normal cats; $^{+}$p<0.05, $^{++}$0.01>p>0.001, $^{+++}$0.001<p<0.0001 vs. untreated SD cats; ^0.05>p>0.01, ^^0.01>p>0.001 vs. low dose at same age. $^{\#}$p<0.05, $^{\#\#\#}$0.001<p<0.0001 vs. short term at same dose.

bioRxiv preprint doi: https://doi.org/10.1101/2024.11.15.623838; this version posted November 18, 2024. The copyright holder for this preprint (which was not certified by peer review) is the author/funder, who has granted bioRxiv a license to display the preprint in perpetuity. It is made available under aCC-BY 4.0 International license.

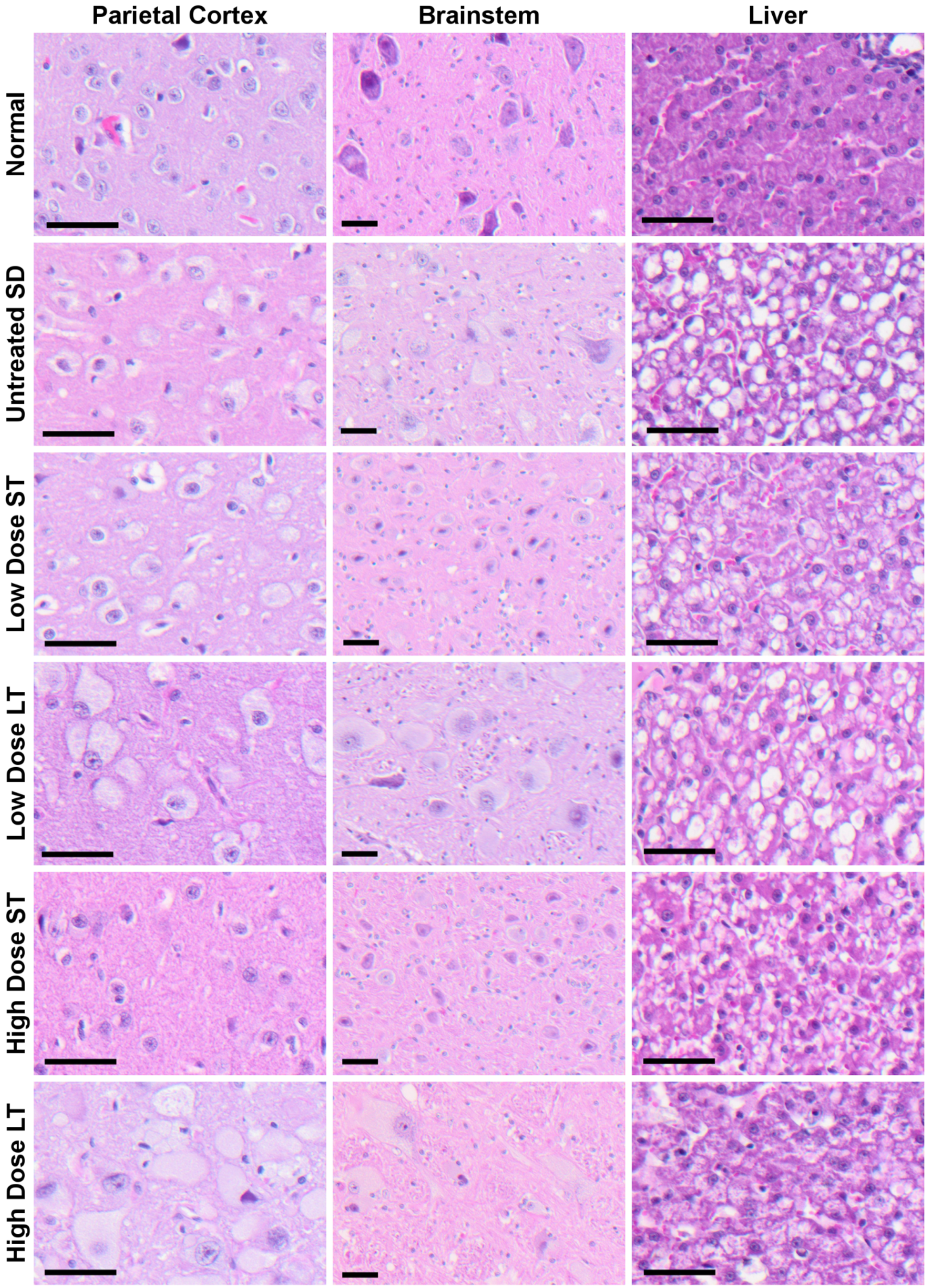

bioRxiv preprint doi: https://doi.org/10.1101/2024.11.15.623838; this version posted November 18, 2024. The copyright holder for this preprint (which was not certified by peer review) is the author/funder, who has granted bioRxiv a license to display the preprint in perpetuity. It is made available under aCC-BY 4.0 International license.

Figure S5. Histopathological storage lesions in neuronal cell bodies and hepatocytes are partially corrected by AAV treatment. Severe storage lesions occur frequently in the neuronal cell bodies and hepatocytes of untreated and AAV treated SD cats with a longer lifespan (long-term groups). Shorter-lived cats treated with the high dose demonstrated near-normalization of these lesions, with cats in the low dose group had moderate improvement. Micrographs at 10x (brainstem) or 20x (parietal cortex, liver) are from the representative cats. Scale bar: 25µm. ST: short-term, LT: long-term

bioRxiv preprint doi: https://doi.org/10.1101/2024.11.15.623838; this version posted November 18, 2024. The copyright holder for this preprint (which was not certified by peer review) is the author/funder, who has granted bioRxiv a license to display the preprint in perpetuity. It is made available under aCC-BY 4.0 International license.

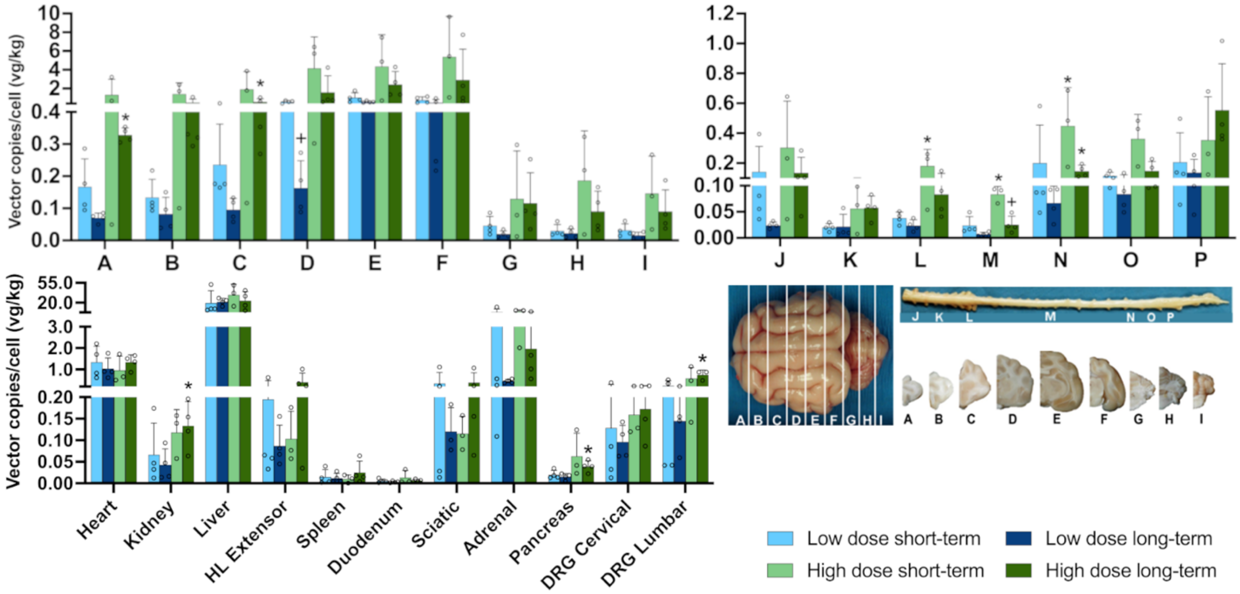

Fig S6. AAV vector biodistribution. AAV vector was distributed throughout the CNS and peripheral tissues, with the highest concentrations in the cerebral cortex and liver. ST: short-term, LT: long-term, HL: hindlimb, DRG: dorsal root ganglion. * indicates $p<0.05$ compared to age-matched low dose cats, + indicates $p<0.05$ compared to short-term cohort given the same dose.

bioRxiv preprint doi: https://doi.org/10.1101/2024.11.15.623838; this version posted November 18, 2024. The copyright holder for this preprint (which was not certified by peer review) is the author/funder, who has granted bioRxiv a license to display the preprint in perpetuity. It is made available under aCC-BY 4.0 International license.

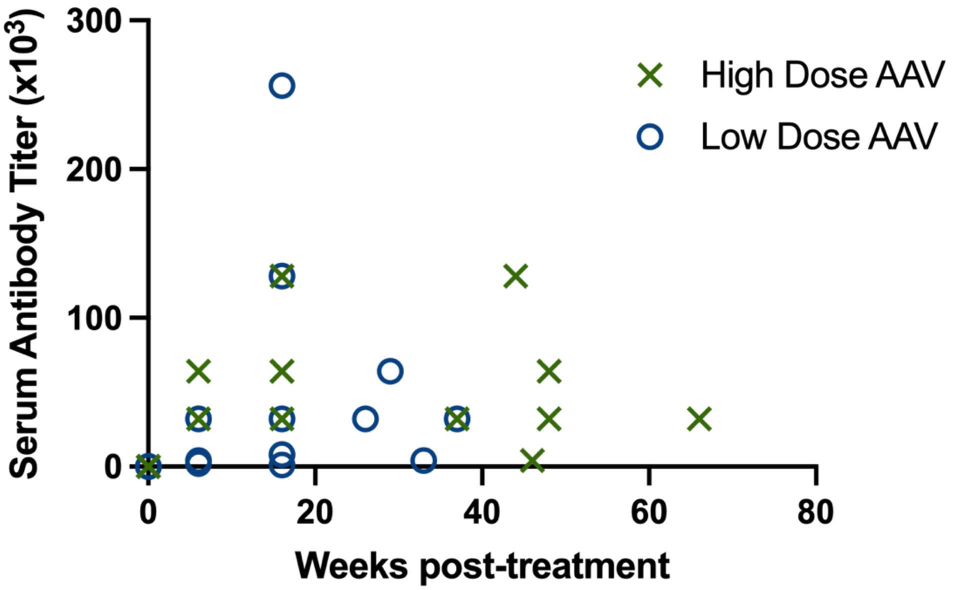

Fig S7. Serum antibody titers in AAV-treated cats. All cats developed antibody titers against AAV9. Overlapping data points within the same treatment group occur at 16 weeks (2 low dose cats with 8000 and 2 high dose cats with 64,000) and are not able to be distinguished on the graph.
